# Thar She Blows! A Novel Method for DNA Collection from Cetacean Blow

**DOI:** 10.1371/journal.pone.0012299

**Published:** 2010-08-25

**Authors:** Céline H. Frère, Ewa Krzyszczyk, Eric M. Patterson, Sue Hunter, Alison Ginsburg, Janet Mann

**Affiliations:** 1 School of Veterinary Sciences, University of Queensland, Brisbane, Australia; 2 Departments of Biology and Psychology, Georgetown University, Washington, District of Columbia, United States of America; 3 National Aquarium, Baltimore, Maryland, United States of America; Texas A&M University, United States of America

## Abstract

**Background:**

Molecular tools are now widely used to address crucial management and conservation questions. To date, dart biopsying has been the most commonly used method for collecting genetic data from cetaceans; however, this method has some drawbacks. Dart biopsying is considered inappropriate for young animals and has recently come under scrutiny from ethical boards, conservationists, and the general public. Thus, identifying alternative genetic collection techniques for cetaceans remains a priority, especially for internationally protected species.

**Methodology/Principal Findings:**

In this study, we investigated whether blow-sampling, which involves collecting exhalations from the blowholes of cetaceans, could be developed as a new less invasive method for DNA collection. Our current methodology was developed using six bottlenose dolphins, *Tursiops truncatus*, housed at the National Aquarium, Baltimore (USA), from which we were able to collect both blow and blood samples. For all six individuals, we found that their mitochondrial and microsatellite DNA profile taken from blow, matched their corresponding mitochondrial and microsatellite DNA profile collected from blood. This indicates that blow-sampling is a viable alternative method for DNA collection.

**Conclusion/Significance:**

In this study, we show that blow-sampling provides a viable and less invasive method for collection of genetic data, even for small cetaceans. In contrast to dart biopsying, the advantage of this method is that it capitalizes on the natural breathing behaviour of dolphins and can be applied to even very young dolphins. Both biopsy and blow-sampling require close proximity of the boat, but blow-sampling can be achieved when dolphins voluntarily bow-ride and involves no harmful contact.

## Introduction

Information on kinship or relatedness is not only central to all theories of social evolution [1], [2], but is also critical for understanding the basic biology of a species or population. As a result, molecular techniques are now widely used to address a broad range of questions including those crucial to the management and conservation of wild populations [3], [4], [5]. While dart biopsying has been the most commonly used method for collecting genetic data from cetaceans (e.g. [4], [6]), this method has some drawbacks. Dart biopsying is considered inappropriate for young animals and often comes under scrutiny from ethical boards, conservationists, and the general public. Such concerns were compounded in 2000, when Bearzi reported the death of a common dolphin after the penetration of a biopsy dart [7]. Since this report, a multitude of studies have investigated the impact of dart biopsying on cetaceans (e.g. [8], [9], [10]), and all concluded that this method results only in short-term minimal disturbance to the animals involved. However, identifying alternative genetic collection techniques for cetaceans remains a priority, especially for internationally protected species. Here, we show that a new non-invasive method of data collection, “blow-sampling”, which involves collecting exhalations from the blowholes of cetaceans, can provide much needed genetic data, even for small cetaceans.

In the last decade, a range of less invasive techniques, including skin swabbing [11], [12] and fecal sampling [13], have been explored with the aim of presenting viable alternatives to dart biopsying. However, such techniques have either failed to consistently amplify nuclear DNA [11], [13], or were not ground-truthed [12]. Ground-truthing can be achieved by testing DNA profiles collected by the trial technique against profiles for the same individuals collected using methods that yield high DNA quantities, such as blood or biopsy sampling. This is important as insufficient DNA quantity can lead to several problems when amplifying microsatellite markers, such as non-specific amplification and allelic dropout [14].

Blow-sampling was first attempted by Hogg [15] to examine hormonal and reproductive state in cetaceans, and has thus far, been used to determine the presence and quantification of several reproductive hormones (e.g. [15], [16]). While Trout [17] has critiqued Hogg *et al*. 's chemical analysis and results [16], he also provided critical suggestions for the improvement of this technique. The relative ease with which blow can be collected and the promise that blow samples may be chemically analysed to answer a number of biological questions have already led several researchers to begin collecting blow samples (e.g. [18]). More recently, blow has also been successfully used to assess disease in free ranging cetaceans [19]. To date however, we are unaware of any published research that has investigated whether blow sampling could be successfully used to extract DNA information from cetaceans.

Blow is a relatively unexplored substance and thus its composition and full potential as a biological sample remains unexplored. Lung surfactant is likely to be the primary biological fluid in cetacean blow [20]. As cetaceans exhale at tremendous flow rates (bottlenose dolphins at ∼70 liters/sec; [21]) it is likely that some respiratory fluid and some lung cells will be exhaled. The chemical makeup of blow is therefore likely to be similar to the composition of the lung surfactant of cetaceans (97% lipid, 2.3% protein, and less than 1% carbohydrate[22]). It may also contain airway secretions, believed to be derived from alveolar surfactant used for airway stabilization [23]. Thus, it is likely that blow is a mix of several types of biological fluids and may hold the answers to numerous biological questions.

In a pilot study, we first investigated whether or not blow samples could be collected from wild bottlenose dolphins in the eastern gulf of Shark Bay, Western Australia (25°47′S,113°43′E). Initially, we used a modified embroidery hoop with sterile filter paper stretched over its centre to collect the blow. We anticipated that absorbent filter paper would maximize sample collection. However, while this was the case, the filter paper inhibited successful DNA extraction from the sample. We did, however, successfully extract DNA from one individual, but were only able to amplify mitochondrial DNA. This preliminary work confirmed not only that blow could be successfully collected from small wild cetaceans, but that also these samples could be used to extract DNA information with appropriate methodological optimisation.

**Figure 1:**
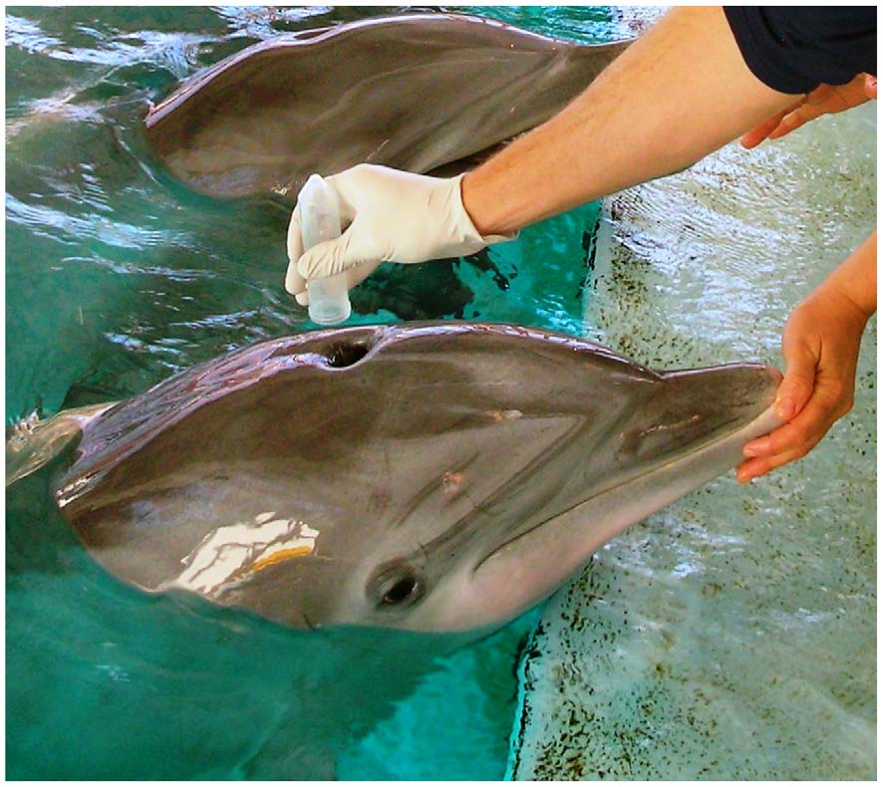
Blow sample collection using sterile 50 ml polypropylene tubes. A test tube was held inverted over the dolphin's blowhole and the dolphins were trained to exhale on cue (Figure 1). Four to six exhalations were collected per tube. We collected two sets of blow samples from each individual using sterile 50 ml polypropylene tubes (Fisherbrand) to test two different storage and transportation methods (detailed below). Both sets of blow samples were stored on dry ice for transportation to Georgetown University (Washington DC, USA). Once in the laboratory, the first set of blow samples were resuspended in 1 ml of 100% ethanol. The sides were scraped down using sterilized policemen, vortexed, and finally centrifuged at 3000 rpm for 3 minutes. Samples were then transferred to 1.5 ml microcentrifuge tubes to dry off the ethanol in a speed vacuum. The second set of blow samples were resuspended in 500 µl of TE buffer and centrifuged at 3000 rpm for 3 minutes. Excess TE buffer was carefully removed from all samples leaving a small amount at the bottom of the tube. DNA extraction of both sets of blow samples and blood samples were completed using a Qiagen DNeasy Blood and Tissue kit, using the animal tissues spin-column protocol. After DNA purification, blow samples were stored at −80°C until transportation to the University of Queensland (Brisbane, Australia) for DNA fingerprinting. A control sample of seawater was taken along with each blow sample set to assure that any DNA results were from blow samples and not seawater contamination.

Our current methodology was developed using bottlenose dolphins, *Tursiops truncatus*, housed at the National Aquarium, Baltimore (USA), from which we were able to collect both blow and blood samples in order to ground-truth our study. All research was approved and permitted through the Georgetown University (Washington DC, USA) Animal Care and Use Committee (GUACUC permit #07-041), as well as through the National Aquarium (Baltimore, USA) Biological Programs Research Committee. Funding was provided by Georgetown University and the National Science Foundation grant number 0847922. Blow (Figure 1) and blood samples were collected between March and May, 2010 from a total of 6 bottlenose dolphins: 5 female bottlenose dolphins (4 adults and 1 sub-adult) and 1 male juvenile. Blood samples were collected as part of each individual's routine quarterly medical examination. Here, we present the results and discuss future directions.

## Results and Discussion

**Figure 2:**
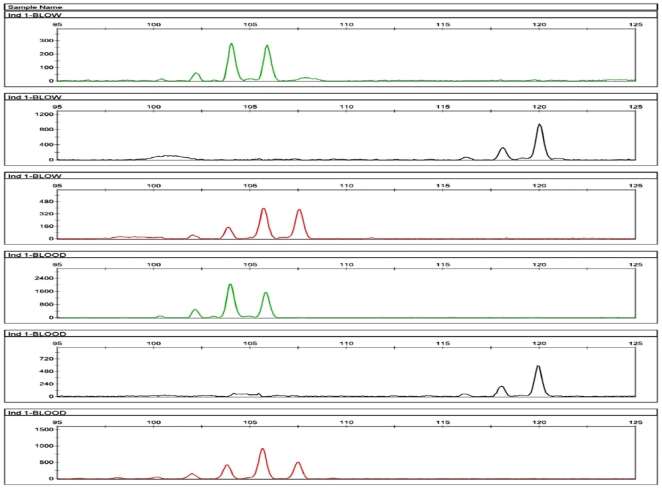
Microsatellite DNA profile of blow and blood from individual 1. To estimate whether the microsatellite profile collected from an individual's blow matched the microsatellite profile collected from its blood, we amplified 3 polymorphic dinucleotide microsatellite loci: Lobs_Di9 (black), Lobs-Di19 (red), and Lobs_Di21 (green) [24]. The three loci were amplified using a Qiagen Multiplex KitTM (Qiagen). The annealing temperature was set at 53°C for 60 seconds for a total of 30 cycles. PCR products were then run on an ABI 3730 DNA Sequencer (Applied Biosystems). Microsatellite profiles were visualised using the software GeneMapper (version 4.0). For ease of presentation, we only include here a figure showing the microsatellite DNA profile of blood and blow for one individual. The remaining 5 microsatellite DNA profiles are included in supporting online information Figure S1. The top 3 panels represent the three microsatellite loci amplified from DNA extracted from blow.

**Figure 3:**
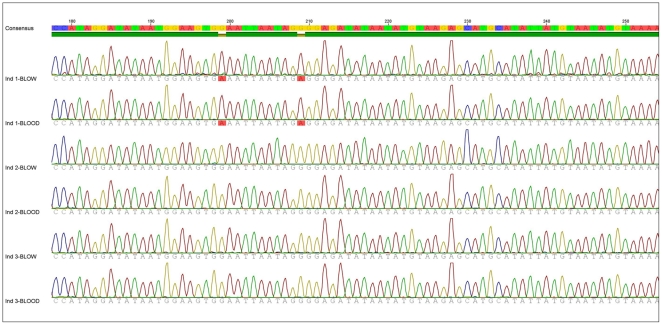
Mitochondrial DNA profile of blow and blood from individuals 1 to 3. To estimate whether the mitochondrial DNA profile collected from an individual's blow matched the mitochondrial DNA profile collected from its blood, we amplified a 426 base-pair fragment of the maternally inherited mitochondrial DNA control region using the primers dlp1.5 [25] and dlp5 [26]. The PCR products were cleaned using ExoSAP-IT from Affymetrix. Cycle sequencing was conducted with the BigDyeTM Terminator Cycle Sequencing Ready Reaction kit (Applied Biosystems). After a magnesium sulphate (MgSO4) clean-up, the sequencing fragments were run on an ABI 3730 DNA Sequencer (Applied Biosystems). The sequences were visualised and edited using Geneious Pro version 5.0. Out of the 434 base-pair fragments amplified across the seven individuals, we identified only two polymorphic sites. As a result and for ease of presentation, we only include here a figure showing an 80 base-pair fragment including the two polymorphic sites for three of the six individuals (178 bp-255 bp). The remaining three individuals' mtDNA profile is available in the supporting online information Figure S2. The full sequences for all six individuals have been made available on GenBank (HM581690-HM58701).

For all six individuals, we found that their mitochondrial and microsatellite DNA profile taken from blow, matched their corresponding mitochondrial and microsatellite DNA profile collected from blood (see Figure 2 & Figure 3). Furthermore, there was no contamination in seawater samples. Having trialled two DNA storage buffers, we recommend resuspending blow samples in TE buffer rather than in ethanol for two reasons. First and foremost, TE buffer will prevent nucleases from degrading DNA present in the sample. EDTA, an active component of TE buffer, will deactivate nucleases by binding to metal ions. Second, we found that blow samples resuspended in ethanol had a tendency to stick to the side of the collection tube, making it more difficult to centrifuge. In addition, we found that DNA extractions of blood and blow yield similar DNA concentration (∼10 ng/µl).

Overall, our study shows that DNA can be extracted from blow samples and that both mitochondrial and nuclear DNA can be successfully amplified using these samples. By comparing the DNA profile of blow to the DNA profile of its corresponding blood sample, we were able to demonstrate that DNA extracted from blow did not lead to problematic microsatellite amplification, such as non-specific amplification and allelic dropout [14]. While this work was conducted using dolphins from aquaria, its application in natural populations is nevertheless promising. Having optimised collection and storage methodology, as detailed above, we are now undertaking blow collection from wild bottlenose dolphins in the eastern gulf of Shark Bay. This population of dolphins has been the focus of extensive study since the mid-1980s [27], [28], making it, along with the Sarasota Florida population (Florida, USA) [29], [30], [31], one of the most comprehensively studied populations of bottlenose dolphins. We have detailed mitochondrial and microsatellite profiles of more than 500 identifiable dolphins in this population which were sampled using projectile biopsy darts [32]. This will allow us to ground-truth blow sampling in this wild population. The advantage of this method is that it capitalizes on the natural breathing behaviour of dolphins and can be applied to even very young dolphins. Both biopsy and blow-sampling require close proximity of the boat, but blow-sampling can be achieved when dolphins voluntarily bow-ride and involves no harmful contact. While we recognise the important role played by dart-biopsying, we provide evidence that blow-sampling is a viable alternative and less invasive mode of DNA collection.

## Supporting Information

Figure S1 Microsatellite DNA profile of blow and blood from the remaining 5 individuals. The top 3 panels represent the three microsatellite loci amplified from DNA extracted from blow. The 3 lower panels represent the three microsatellite loci amplified from DNA extracted from blood. The microsatellite locus lobs_Di21 is coloured green. The microsatellite locus Lobs_Di9 is coloured black. And the microsatellite locus Lobs_Di9 is coloured red.
(4.75 MB TIF)

Figure S2 Mitochondrial DNA profile of blow and blood from individuals 4 to 6. For ease of presentation we only show an 80 base pair long fragment (178 bp-255 bp) of the 434 base pair long sequence for three out of the six individuals. The remaining three DNA profiles are available on the online supporting material Figure S2. Additionally, the full sequences are available on GenBank accession numbers (HM581690-HM58701).
(4.66 MB TIF)
