# Supplementary figures and images for: Thar She Blows! A Novel Method for DNA Collection from Cetacean Blow
Source: PLoS One. 2010 Aug 25;5(8):e12299. doi: 10.1371/journal.pone.0012299 (PMC2928266; doi:10.1371/journal.pone.0012299)

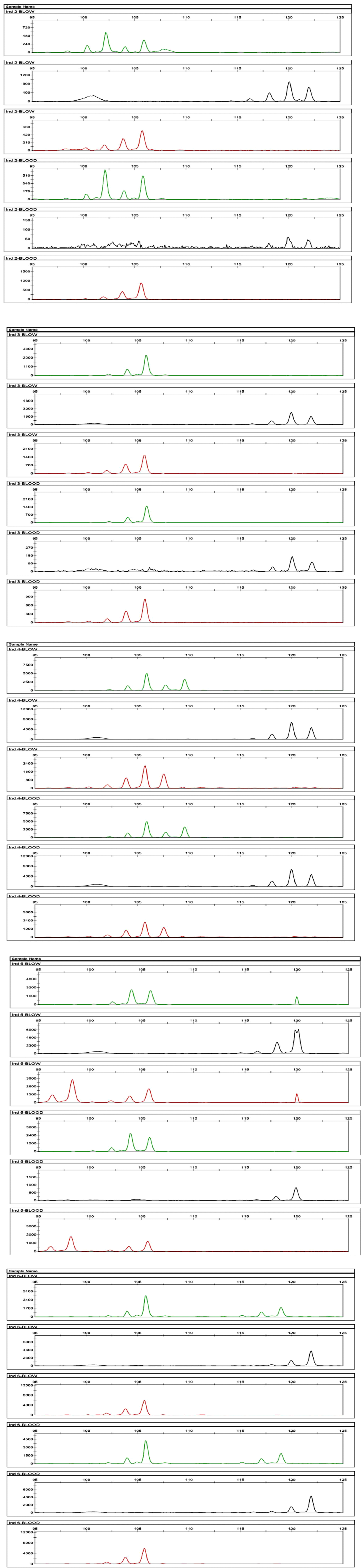

Supplement: Figure S1 — Microsatellite DNA profile of blow and blood from the remaining 5 individuals. The top 3 panels represent the three microsatellite loci amplified from DNA extracted from blow. The 3 lower panels represent the three microsatellite loci amplified from DNA extracted from blood. The microsatellite locus lobs_Di21 is coloured green. The microsatellite locus Lobs_Di9 is coloured black. And the microsatellite locus Lobs_Di9 is coloured red. (4.75 MB TIF) [file pone.0012299.s001.tif]

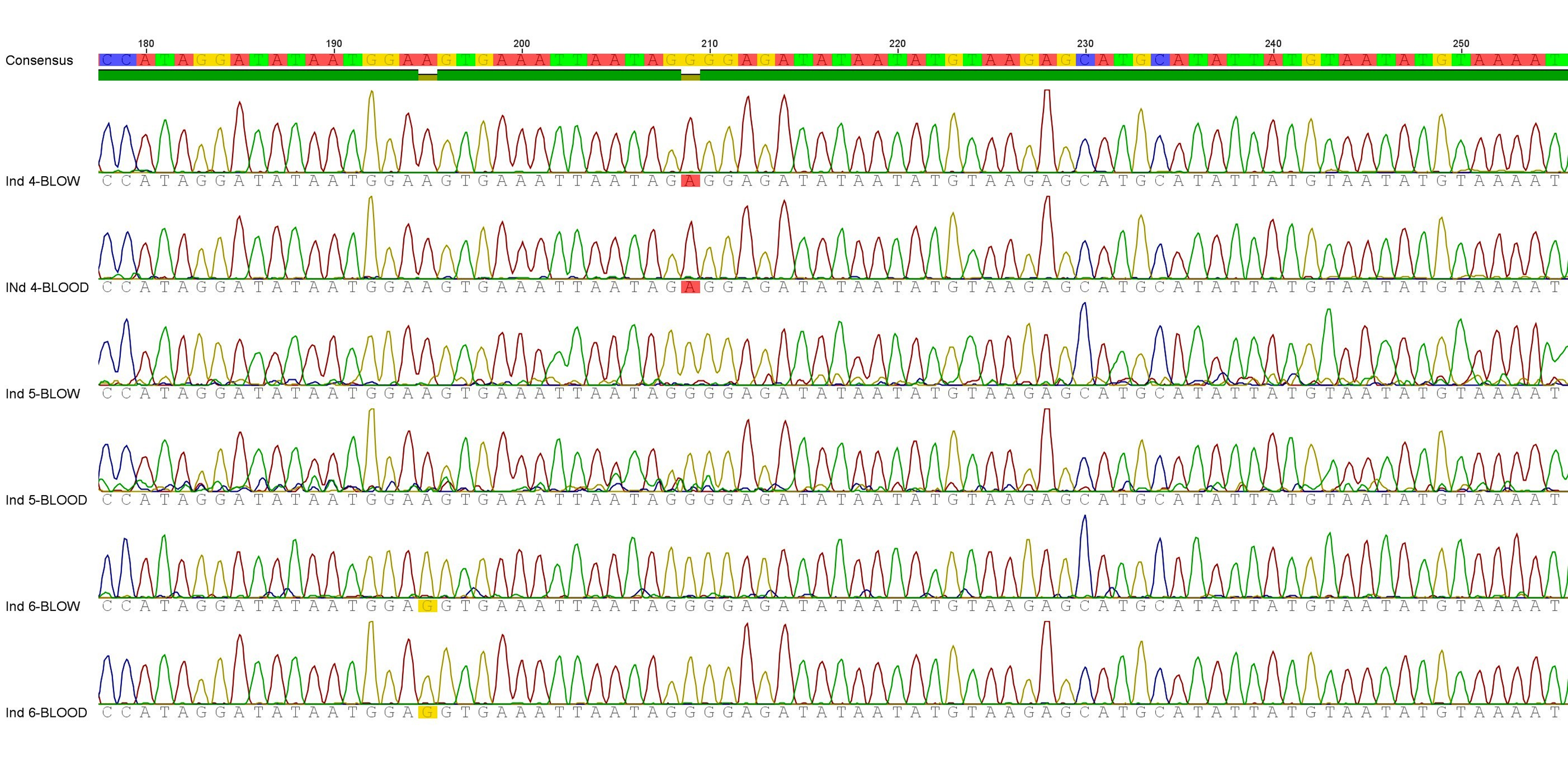

Supplement: Figure S2 — Mitochondrial DNA profile of blow and blood from individuals 4 to 6. For ease of presentation we only show an 80 base pair long fragment (178 bp-255 bp) of the 434 base pair long sequence for three out of the six individuals. The remaining three DNA profiles are available on the online supporting material Figure S2. Additionally, the full sequences are available on GenBank accession numbers (HM581690-HM58701). (4.66 MB TIF) [file pone.0012299.s002.tif]
